# Supplementary material for: The Epidemiological and Economic Impact of COVID-19 in Kazakhstan: An Agent-Based Modeling
Source: Healthcare (Basel). 2023 Nov 16;11(22):2968. doi: 10.3390/healthcare11222968 (PMC10671669; doi:10.3390/healthcare11222968)
Supplement: Supplementary file 1 [file healthcare-11-02968-s001.zip › healthcare-2691149-supplementary.pdf]

Model parameters

Table S1. Social and demographic parameters

Variable	Unit	Current value	References
Population	number	500 896	https://stat.gov.kz (accessed on 1 July 2023)
– children of preschool age	%	3	
– schoolchildren	%	14	
– students	%	7	
– workers	%	61	
– retired	%	15	
Total families	number	173501	
– 1 person family	%	18,9	
– 2 persons family	%	27,2	
– 3 persons family	%	19,1	
– 4 persons family	%	15,9	
– 5 persons family	%	18,9	
Total workers	number of people	301785	
– in enterprises with a staff of up to 100 people	%	20	
– in enterprises with a staff of up to 250 people	%	18	
– in enterprises with a staff of up to 500 people	%	62	
Owners of personal vehicles	%	21	
Occupancy rate for a group in kindergartens	number of people	25	Model rules for the activities of educational organizations https://adilet.zan.kz/rus/docs/V1800017657 (accessed on 1 July 2023)
Group occupancy rate in schools	number of people	25	
Group occupancy rate in university	number of people	25	
Average attendance of one "convenience store"	number of people	50	https://karaganda.adresa-telefony.ru/produktovye_magaziny#: (accessed on 1 July 2023)
Number of clinics	number	7	https://stat.gov.kz (accessed on 1 July 2023)
Average bus capacity	number of people	60	In average

Table S2. Epidemiological parameters

Variable	Unit	Current value	References
Average daily contact	number	6	[49]
Average incubation period	day	6	[50]
Hospitalization rate	%	29	Ministry of Health of the Republic of Kazakhstan
Children hospitalization rate		8,1%	
Case severity			
– mild		74%	
– moderate		23%	
– severe		2%	
– critical (inpatient, ventilation)		1%	
Fatality rate	%	1,1	
Critical fatality rate	%	91	
Duration of outpatient treatment	day	triangular(8,10,14)	
Duration of inpatient treatment	day	triangular(15,20,28)	
Duration of critical care (invasively ventilated)	day	triangular(9,10,14)	

Table S3. Infection by location in the simulated scenarios

Scenario	Total cases	At home	At work	In transport	At univers.	At school	At preschool	In mall
BS	471746	201826 (43%)	135605 (29%)	73682 (16%)	11607 (2%)	24807 (5%)	11177 (2%)	13042 (3%)
SC	438610	218731 (50%)	137590 (31%)	71107 (16%)	0	0	0	11182 (3%)
MV	215715	101481 (47%)	60138 (28%)	32597 (15%)	3574 (2%)	9373 (4%)	5477 (3%)	3075 (1%)
VS	307381	132894 (43%)	91667 (30%)	49289 (16%)	5874 (2%)	14703 (5%)	7416 (2%)	5538 (2%)
CM	604	270 (45%)	215 (35%)	113 (19%)	0	0	0	6 (1%)
RS	145044	N/A	N/A	N/A	N/A	N/A	N/A	N/A

Table S4. Age groups infection transmission comparison

Scenario	Total cases	Age group			
		0-17	18-25	26-59	60+
BS	471746	85604 (18%)	35075 (7%)	292562 (62%)	58505 (12%)
SC	438610	70513 (16%)	28362 (6%)	285168 (65%)	54567 (12%)
MV	215715	36429 (17%)	13860 (6%)	144711 (67%)	20715 (10%)

VS	307381	52036 (17%)	20354 (7%)	203043 (66%)	31948 (10%)
CM	604	63 (10%)	17 (3%)	471 (78%)	53 (9%)
RS	145044	11604 (8%)	10153 (7%)	55117 (38%)	68171 (47%)

Table S5. Hospital beds needed

Scenario	BS	SC	MW	VS	CM	RS
Total number of severe cases needing beds (unconstrained by bed availability)	7454	6930	3408	4857	10	2292
Total number of severe patients admitted and in a bed (capped by bed availability)	4256	4256	3408	4256	10	2292
% Available beds for severe patients that are currently occupied	100%	100%	80%	100%	0%	54%
Number of additional beds needed to treat all severe patients who need them	3198	2675	-	601	-	-
Total number of critical cases needing beds (unconstrained by bed availability)	6699	6228	3063	4365	9	2060
Total number of critical patients admitted and in a bed (capped by bed availability)	202	202	202	202	9	202
% Available beds for critical patients that are currently occupied	100%	100%	100%	100%	4%	100%
Number of additional beds needed to treat all critical patients who need them	6496	6026	2861	4162	-	1857

Table S6. The costs of goods

	BS	SC	MW	VS	CM	RS
Hygiene	$325498	$305174	$163439	$224,683	$665	$109787
PPE	$681549	$655629	$452237	$552,981	$4,204	$351771
Diagnostics	$69840	$69840	$67320	$69,840	$13,088	$63720
Pharmaceuticals	$4614981	$4614,981	$3776023	$4,614,981	$26,438	$2588645
Biomedical Equipment & Non-consumables	$39417714	$39417,714	$32972178	$39,417,714	$370,944	$23849801
Biomedical Consumables & Accessories	$65392	$65392	$56682	$65,392	$1,015	$44356
TOTAL	$45174975	$45128731	$37487881	$44945592	$416354	$27008080
